# Patient-reported outcome after hip dislocation in primary total hip arthroplasty is virtually unknown: a systematic literature review

**DOI:** 10.1080/17453674.2018.1518428

**Published:** 2018-10-17

**Authors:** Lars L Hermansen, Martin H Haubro, Bjarke L Viberg, Søren Overgaard

**Affiliations:** 1 Department of Orthopaedics, Hospital of South West Jutland, Esbjerg;;; 2 The Orthopaedic Research Unit, Department of Orthopaedic Surgery and Traumatology, Odense University Hospital, Odense;;; 3 Department of Clinical Research, Odense University Hospital, Odense;;; 4 Department of Orthopaedic Surgery and Traumatology, Kolding Hospital, Kolding;

Most patients have good to excellent outcomes after total hip arthroplasty (THA) due to osteoarthritis (OA). However, severe complications do still occur, and hip dislocation remains one of the most common reasons for revision surgery in the first postoperative years (Bozic et al. 2015, Singh et al. 2016). The incidence of hip dislocation after primary THA ranges from 1% to 10% (Dargel et al. 2014, Jorgensen et al. 2014, Petis et al. 2015, Zhang et al. 2015).

Implant malposition or loosening is an obvious reason for surgical intervention after hip dislocations. In cases with no clear etiology, the non-surgical treatment is often prolonged, and the effect of the dislocation on daily activities and the subjective hip symptoms become more essential. The outcome after revision surgery due to recurrent dislocations is also not encouraging, as 10–34% of the revised patients re-dislocate (Wetters et al. 2013, Jo et al. 2015, Yoshimoto et al. 2017).

In order to advise these patients properly, it is important to know the impact of 1 or recurrent dislocations on the patient’s quality of life and self-experienced hip function. This will contribute to an improved decision-making process for the patients, with no obvious cause for their hip dislocation. Thus, we conducted a systematic review of studies comparing patient-reported outcomes (PROs) in patients with a primary THA due to OA with and without episodes of hip dislocation.

## Method

This review is reported with respect to the Preferred Reporting Items for Systematic reviews and Meta-Analysis (PRISMA) statements (Moher et al. 2009). The protocol was based upon the Preferred Reporting Items for Systematic reviews and Meta-Analysis Protocols (PRISMA-P) guidelines (Moher et al. 2015) and registered in the International Prospective Register of Systematic Reviews (PROSPERO) database (CRD42017076125).

We searched Pubmed, Embase, SveMed, and Cochrane databases for relevant literature from the origin of each database and up to September 1, 2017. The reference list in each of the included studies was scanned for additional eligible studies.

Studies were included in the review if the following criteria were fulfilled:

- *Study designs*: randomized controlled trials (RCT), non-randomized controlled clinical trials, prospective/retrospective cohort, and case-control studies (level of evidence 1–3).
- *Participants*: OA as a primary diagnosis.
- *Outcomes*: PRO after dislocation, either completely patient-reported measurements or measures, where the patient part was reported separately from the clinician’s evaluation.
- Studies published in English, German, and Scandinavian language.
- No “prior to revision surgery” studies were included (selected patients).

Risk of bias within cohort and case-control studies were assessed using the Critical Appraisal Skills Programme (CASP 2017) tool for relevant study designs. We performed the quality assessment of the eligible studies before data were extracted and this was carried out by 2 reviewers.

## Results

We identified 3,460 unique studies using our broad search query. Throughout the title/abstract screening, 3,432 studies were excluded. We assessed full text of the remaining 28 articles for eligibility and, of these, only 2 studies (Forsythe et al. 2007, Edmunds and Boscainos 2011) met the inclusion criteria. No further studies were identified after screening the reference lists of the 2 included studies.

The study by Forsythe et al. reported no statistically significant differences in the reduced WOMAC and SF-12 scores between the 32 patients with dislocation and the 64 patients without dislocation. However, the group without dislocation was significantly more satisfied postoperatively. Edmund and Boscainos primarily compared the anterolateral and posterior approaches. The combined results from patients with or without dislocations were not presented. The authors simply concluded that patients with dislocation lose approximately 5 points in total HHS, compared with non-dislocators. 3 of these points were represented by the function score since the HHS is only partly patient reported. No statistics were performed.

A meta-analysis was not possible, since 4 different patient­reported outcome measures (PROMs) had been used in the 2 included studies. Since only 2 studies met the inclusion criteria, comparing PROs in THA patients with/without hip dislocation, we aimed to extend the scope of the present review, and also to present papers covering PROM after dislocation, without comparisons. However, we found no additional studies presenting PROMs after a dislocation episode exclusively in THA patients with OA as the primary diagnosis.

## Discussion

The goal of this systematic review was to provide valuable information regarding patient experience after dislocating a primary THA. Our review revealed that knowledge of patient-reported quality of life and subjective hip function after dislocation is merely non-existent. Efforts are ongoing to raise the use of PROMs in orthopedics from study to registry level across Europe. This will enable future prospective studies to evaluate the subjective importance of various complications (Paulsen 2014, Rolfson et al. 2016). A challenge though, is that closed reduction of prosthesis dislocation without revision is not reported in hip registries.

Presumably, quality of life must be affected and continuously decreases with recurrent events. Likewise, confidence and trust in hip function and stability is impaired. These statements need scientific support. We are planning a larger scale study to identify differences in PRO for patients with single and recurrent dislocations.

The original paper is unpublished and available on request to the corresponding author.
